# Lycopene as A Carotenoid Provides Radioprotectant and Antioxidant Effects by Quenching Radiation-Induced Free Radical Singlet Oxygen: An Overview

**DOI:** 10.22074/cellj.2015.485

**Published:** 2015-01-13

**Authors:** Jalil Pirayesh Islamian, Habib Mehrali

**Affiliations:** Department of Medical Physics, School of Medicine, Tabriz University of Medical Sciences, Tabriz, Iran

**Keywords:** Antioxidant, Carotenoid, Free Radical, Lycopene, Radioprotectant

## Abstract

Radio-protectors are agents that protect human cells and tissues from undesirable effects of ionizing radiation by mainly scavenging radiation-induced free radicals. Although chemical radio-protectors diminish these deleterious side effects they induce a number of unwanted effects on humans such as blood pressure modifications, vomiting, nausea, and both local and generalized cutaneous reactions. These disadvantages have led to emphasis on the use of some botanical radio-protectants as alternatives. This review has collected and organized studies on a plant-derived radio-protector, lycopene. Lycopene protects normal tissues and cells by scavenging free radicals. Therefore, treatment of cells with lycopene prior to exposure to an oxidative stress, oxidative molecules or ionizing radiation may be an effective approach in diminishing undesirable effects of radiation byproducts. Studies have designated lycopene to be an effective radio-protector with negligible side effects.

## Introduction

Humans are exposed to ionizing radiation; thus they are potentially at increased risk for adverse health effects. At-risk populations include diagnostic and therapeutic ionizing radiation workers, victims of nuclear fallout and nuclear terrorism, workers in the nuclear power industry, waste clean-up crews, residents of places in close proximity to nuclear plants or research laboratories with radiological facilities, patients undergoing routine diagnostic or therapeutic radiation treatment procedures, astronauts occupationally exposed to cosmic radiation and members of the armed forces that are potentially subjected to intentional sources of radiation (1). Exposure to higher doses of ionizing radiation (above some cGy), however, results in increased rates of genetic mutations and cell death (2). Radiation-induced damages can be severe when the biological environment is exposed to radiosensitizers (3, 4). Radioprotectants are important in protecting cells from deleterious radiation-induced side effects (5, 6).

Chemically produced radio-protectors diminish these side effects but may also induce a number of unwanted effects on humans such as blood pressure modifications, vomiting, nausea, and local and generalized cutaneous reactions (7-9). These disadvantages have led to improvements in botanical radio-protectants as alternatives (10-12).

### Carotenoids as botanical radioprotectors

Carotenoids are present at considerable amounts in plasma and human tissues and may have specific functions in relation to their high antioxidant capacity (13). Carotenoids have been found to decrease the potential stress of reactive oxygen species (ROS) within aerobic metabolism (14, 15). Lycopene, as a bright red carotene, is the main carotenoid present in tomatoes and other red fruits and vegetables including red carrots, red bell peppers, watermelons, gac, and papayas but not strawberries or cherries. *In vitro* studies have shown that lycopene, a polyunsaturated hydrocarbon with a molecular formula of C_40_H_56_, has the highest antioxidant capacity to quench singlet oxygen and trap peroxyl radicals (16, 17). Although lycopene is chemically a carotene it has no vitamin A activity. Tomato products have higher levels of antioxidant activity and therefore are more potent than tomatoes in reducing the risk of oxidation-related diseases (18-21). Tomatoes contain a number of different compounds such as carotenoids, vitamin C, and flavonoids that may account for its antioxidant properties. However lycopene, as the main carotenoid in tomato products, possesses the greatest ability to quench singlet oxygen compared to the other carotenoids (22). The lycopene molecule is long and straight, and constrained by its system of 11 conjugated double bonds. Lycopene cyclase is an enzyme found in tomatoes that can convert lycopene to β-carotene by catalyzing the formation of two β-rings at each end of the linear carotene. Eleven conjugated double bonds of lycopene give it a deep red color and are responsible for its antioxidant activity. Increased ingestion of tomatoes and tomato products that contain lycopene is associated with decreased risk of chronic diseases including cancer (18, 19). For example, there is an inverse relation between serum and tissue lycopene levels to prostate cancer risk; at higher lycopene concentrations, a lower risk of prostate cancer is observed (20, 23).

### Antioxidant and radioprotective effects of lycopene

Oxidative stress is considered one of the major factors connected with increased cancer risk. Lycopene has been found to be the most potent antioxidant among various common carotenoids (22). Lycopene can trap singlet oxygen and reduce mutagenesis in the Ames test. The antioxidant activity of carotenoids in multilamellar liposomes has been assayed by inhibition of formation of thiobarbituric acid-reactive substances (24). Lycopene is the most potent antioxidant among pigments (e.g. from the most potent to the least potent antioxidants are: lycopene, α-tocopherol, α-carotene, β-cryptoxanthin, zeaxanthin=β-carotene, and lutein) (25). In a study conducted by Forssberg et al. (26), it has been shown that lycopene, when injected intraperitoneally as a particulate suspension in irradiated mice, had moderate curative action when administered both before and after an x-ray dose. The researchers have reported increased survival rates. Lymphocytes are good markers of the actual body state and may be a reliable model for studying the effect of additions of specific antioxidants to the diet (13, 27, 28). It has shown that lycopene is effective in protecting blood lymphocytes from NO_2_ radical damage (29). DNA damage is a useful biomarker of the oxidative status and the antioxidant defense system (27). DNA damage in primary lymphocytes was induced *in vitro* by H_2_O_2_ and its effect measured by the Comet assay. Duthie et al. (27) studied a high concentration of H_2_O_2_ (500 μmol/L) to intensify DNA damage and highlighted cells that were able to protect themselves from the resultant oxidative stress. In this research, it was shown that DNA damage of lymphocytes challenged with H_2_O_2_ was reduced by 50% after subjects consumed tomato puree for 14 days. This result was attributed to improvements in cell antioxidant capacity. Deterioration analysis showed a strong inverse relation between plasma lycopene concentration and lymphocyte DNA damage. Therefore, with a consistent dietary antioxidant intake, plasma antioxidant concentrations determined cellular antioxidant capacity (30). Porrini et al. (31) demonstrated in their study on lycopene plasma uptake that total plasma lycopene concentrations increased by 0.5 μmol/L after the first 21-day experimental period in the group that consumed a tomato diet and decreased by 0.2 μmol/L in the group that consumed a tomato-free diet. Another study showed decreased lymphocyte DNA damage after *in vitro* treatment with hydrogen peroxide in groups that consumed a tomato diet (13). Daily consumption of 60 g of tomato puree for 3 weeks has been shown to increase lycopene and ß-carotene plasma concentrations and enhance the resistance of lymphocyte DNA to oxidative stress (32). Some studies have described the carotenoid plasma response after tomato product intake however, little is known about the relationship between the intake of foods rich in carotenoids and their concentrations in specific cells or the amount necessary to ensure antioxidant activity. Reports of supplementation with pure substances such as ß-carotene exist (32-34). Murata et al. (35) have found that ß-carotene concentrations in plasma, peripheral blood monocytes and platelets from subjects supplemented with 60 mg ß-carotene for 44 weeks were higher than in subjects who were given a medication. However, there were no differences observed in red blood cells. They found that the daily consumption of 25 g tomato puree for 14 days significantly increased plasma and lymphocyte lycopene concentrations, whereas the ß-carotene concentration increased only in plasma, and the other carotenoids remained constant (35). The amount of lycopene consumed in the form of tomato was quite low and the period of intake short. However the bioavailability of lycopene from tomato puree was probably very high, which resulted in a significant increase not only in plasma but also in the cells (31). Previously, Porrini et al. (31) have reported an increase in plasma lycopene concentration of 0.5 μmol/L after consumption of 60 g of tomato puree daily for 3 weeks and an increase to 0.4 μmol/L after consumption of less than half that quantity of tomato puree for 2 weeks. Consequently, the lower amounts seemed sufficient to improve and maintain plasma levels. Hence plasma lycopene concentrations were not dose-dependent. The study also showed that 25 g of tomato puree was sufficient to improve lycopene concentrations even in lymphocytes, where they found approximately a doubling effect (32).

The radioprotective effects of aqueous extract of tomato extract (lycopersicon esculentum) was studied by Dhirhe et al. (36) who researched chromosomal aberrations in bone marrow cells of irradiated mice. Healthy adult Swiss mice were injected intraperitoneally with 480 mg/kg or 960 mg/kg body weight of the extract 30 minutes before whole body exposure to 2 and 4 Gy gamma radiation. The results showed that radiation (4 Gy) increased the number of aberrant cells from less than 1% in controls to 20% in the test group. In this study, it was demonstrated that pre-treatment with the extract compounds resulted in a significant reduction in the percentage of aberrant metaphases as well as in the different types of aberrations scored. Hypochlorous acid (HOCl) is an oxidant linked to tissue oxidation in cardiovascular disease and other inflammatory disorders through its ability to modify proteins, deoxyribonucleic acid, ribonucleic acid and lipids (37). Although there is significant evidence that supports the action of lycopene as a potent antioxidant, there are a number of other potential mechanisms such as intercellular gap junction communication, hormonal and immune system modulation and metabolic pathways through which tomato products containing lycopene and other phytochemicals may reduce the risk for chronic diseases, including common forms of cancer and heart disease (38). The ability of carotenoids to induce gap junction communication between cells has also been suggested as a potential basis for their protective effects against cancer development. Lycopene has the capability of improving cell-to-cell communication, but its effects are less pronounced compared to pcaroteneor canthaxanthin (28). A recent study has reported differential dose-related effects of pcarotene and lycopene on gap junction communication in the rat liver. Very low doses (0.5 mg/kg b.w.) had no effect whereas a medium dose (5 mg/kg b.w.) was enhancing and a very high dose (50 mg/kg b.w.) inhibited gap junction communication in the rat liver *in vivo* (39). It appears that gap junction communication ability of carotenoids and their singlet oxygen quenching abilities or antioxidant properties are independent of each other (40). Lycopene acts as a moderate hypocholestremic agent and this effect is related to the inhibition of 3-hydroxy-3-methyl glutaryl coenzyme A (HMGCoA) reductase, the rate limiting enzyme in cholesterol synthesis (41). Lycopene has also been suggested to have modulating effects on liver drug metabolizing enzyme cytochrome P-450 2El (42).Therefore, lycopene can act as a potent scavenger of HOCl at a wide range of concentrations. High-performance liquid chromatography (HPLC), liquid chromatography (LC) and mass spectrometry (MS) analyses have shown that exposure of lycopene to increasing concentrations of HOCl gave a range of metabolites that resulted from oxidative split of one or more C=C in the compound. The degree of degradation of lycopene (as assessed by the number and chain lengths of the various oxidative metabolites of lycopene) depended mainly on the ratio of HOCl to lycopene, which suggested that multiple molecules of HOCl were consumed per molecule of lycopene. Collectively, this study demonstrated a direct link between lycopene and HOCl scavenging and might assist in explaining the mechanism of protective function exerted by lycopene (13). A study conducted by Srinivasan et al. (43) about the effects of irradiation at different doses (1, 2 and 4 Gy) on lymphocytes resulted in a significant increase in the number of micronuclei (MN), dicentric (DC) and translocation frequency. The maximum damage to lymphocytes was observed at 4 Gy of irradiation. However, the frequency of MN, DC and translocation decreased following pretreatment by lycopene. The 5 μg/ml dose of lycopene was found to be more effective. They have concluded that pretreatment with lycopene offers protection to normal lymphocytes against radiation-induced cellular damage (43). Lycopene is a non-chemical antioxidant with minimal side effects, it is abundant in tomatoes and easily available at a low price, all of which make lycopene a favorable radio-protectant (13, 44).

## Discussion

Epidemiologic studies have demonstrated the importance of consuming fruits and vegetables for the preservation of health. Carotenoids are present in considerable amounts in fruits and vegetables (13). There is a clear evidence from epidemiologic studies that intake of 400-600 g per day of fruits and vegetables is associated with a reduced risk of common aero digestive cancers and increased serum á-carotene and lycopene levels are associated with a reduced risk of lung cancer, even among smokers (45). The protective effect has been shown after consumption of products that contain different carotenoid bioavailabilities, such as tomato puree, raw tomatoes and tomato sauce (31, 46). In several studies carotenoid concentrations have been observed in cells obtained from tissue biopsies (47, 48). In an interesting report published by Chen et al. (47), 32 patients with localized prostate adenocarcinoma consumed tomato sauce-based pasta dishes for 3 weeks (30 mg lycopene/day) prior to undergoing radical prostatectomies. Following the dietary intervention, lycopene concentrations in their serum doubled and increased from 0.28 to 0.82 nmol/g in their prostate tissue. Of note, Bhosle et al. (49) found lower oxidative DNA damage both in the prostate tissue and leukocyte samples from their subjects, which suggested that leukocytes could be a surrogate marker to monitor the effectiveness of antioxidant interventions (49). Ionizing radiation has been shown to induce oxidative stress through generation of ROS resulting in an imbalance of the pro-oxidant and antioxidant in the exposed cells (50). Pretreatment of lycopene to ã-irradiated lymphocytes resulted in decreased lipid peroxidation and improved antioxidant status which prevented damage to the lymphocytes. This might be attributed to the antioxidant sparing action of lycopene. Lycopene inhibits cisplatin induced lipid peroxidation in rat testes (51). A possible mechanism by which carotenoids quench singlet oxygen and other excited species occurs when during singlet oxygen quenching, energy is transferred from singlet oxygen to the lycopene molecule converting it to an energy-rich triplet state (51). Trapping of other ROS, OH, NO_2_ or peroxynitrite leads to oxidative breakdown of the lycopene molecule. Thus, lycopene may protect *in vivo* against oxidation of lipids, proteins and DNA (52). Recent studies have shown that daily consumption of tomato products significantly reduces DNA damage from treatment with Fe²+, providing further evidence that this type of dietary intervention improves protection from different oxidative species such as H_2_O_2_, NO_2_, UV and transition metal ions (53, 54). Lycopene is the main carotenoid present in tomatoes and tomato products. Studies show that antioxidant capacity is improved by consumption of tomato products, thereby decreasing the risk of the development of diseases related to oxidative stress (18-21). Bendich and Olson (14) and Britton (15) have shown that carotenoids decrease the potential stress caused by ROS produced by aerobic metabolism. A study by Di Mascio et al. (22) confirmed that lycopene has the highest antioxidant capacity of the carotenoids, having the ability to quench singlet oxygen and trap peroxyl radicals. A number of studies showed that daily consumption of tomato puree increased lycopene plasma concentrations and enhanced the resistance of lymphocyte DNA to an oxidative stress (32, 35). An approximately doubling effect and strong inverse relation between plasma lycopene concentration and lymphocyte DNA damage in addition to decreased chromosome aberration following used of intraperitoneal injection of aqueous tomato extract prior to whole body irradiation at a dose of 4 Gy gamma radiation was shown (13, 27, 28, 30, 43). Pennathur et al. (37) have found a direct link between lycopene and HOCl scavenging which may assist in explaining the mechanism of the protective function used by lycopene. According to the literature, lycopene as the main carotenoid present in tomato and tomato products plays a key role as an antioxidant and a radioprotective substance. At the cellular level, pretreatment with lycopene protects lymphocytes from gamma radiation-induced damage by inhibiting peroxidation of membrane lipids and free radicals which induce the formation of DNA strand breaks.

## Conclusion

Radioprotectants protect against the deleterious effects of ionizing mainly by scavenging by-products from the biological environment. Induced side effects of chemical radioprotectants cause improvement in botanical radioprotectants. According to the related studies, lycopene, as an effective scavenger may be used with negligible side effects.
